# Changes in biomarkers in equine synovial fluid two weeks after intra-articular hyaluronan treatment: a randomised double-blind clinical trial

**DOI:** 10.1186/s12917-018-1512-2

**Published:** 2018-06-15

**Authors:** Tytti M. Niemelä, Riitta-Mari Tulamo, Kaisa Aaltonen, Satu M. Sankari, Anna K. Hielm-Björkman

**Affiliations:** Department of Equine and Small Animal Medicine, Faculty of Veterinary Medicine, University of Helsinki, P.O. Box 57, 00014 Helsinki, Finland

**Keywords:** Aggrecan chondroitin sulfate 846 epitope, Biomarkers, Carboxypeptide of type II collagen, Clinical study, Non-animal stabilized hyaluronic acid (NASHA), Placebo-controlled, Prostaglandin E_2_, Substance P

## Abstract

**Background:**

Inflammatory and degenerative activity inside the joint can be studied in vivo via analysis of synovial fluid (SF) biomarkers, which are molecular markers of inflammatory processes and tissue turnover. The aim of this study was to investigate the response of selected biomarkers in the SF after an intra-articular (IA) high-molecular-weight non-animal stabilized hyaluronic acid (NASHA) treatment. Our hypothesis was that prostaglandin E_2_ (PGE_2_), substance P, aggrecan chondroitin sulfate 846 epitope (CS846), and carboxypeptide of type II collagen (CPII) concentrations in SF would decrease more in the NASHA than in the placebo group. Twenty-eight clinically lame horses with positive responses to diagnostic IA anaesthesia of the metacarpophalangeal or metatarsophalangeal joints were randomized into treatment (*n* = 15) and control (*n* = 13) groups. After collection of baseline SF samples followed by IA diagnostic anaesthesia, horses in the treatment group received 3 ml of a NASHA product IA. Those in the placebo group received an equivalent volume of sterile 0.9% saline solution. The horses were re-evaluated and a second SF sample was obtained after a 2-week period.

**Results:**

CS846 concentration decreased in the NASHA group only (*P* = 0.010). Both PGE_2_ and CPII concentrations decreased within the groups (PGE_2_, *P* = 0.010 for the NASHA group; *P* = 0.027 for the placebo group; CPII, *P* < 0.001 for NASHA group; *P* = 0.009 for placebo group). No significant treatment effect for any biomarker was found between groups. NASHA induced an increase in white blood cell count; this was significant compared with baseline (*P* = 0.021) and the placebo group (*P* = 0.045).

**Conclusions:**

Although the SF concentration of the cartilage-derived biomarker CS846 decreased in the NASHA group, no statistically significant treatment effect of any of the biomarkers were observed between treatment groups. The significant increase in SF white blood cell count after IA NASHA may indicate a mild inflammatory response. However, as no clinical adverse effects were observed, we conclude that IA NASHA was well tolerated.

## Background

A majority of athletic horses suffer from lameness during their careers. Lameness resulting from joint disease is one of the major causes of poor performance [1, 2]. Intra-articular (IA) hyaluronan (HA) injection is a commonly used treatment for equine synovitis and osteoarthritis (OA), although only a few clinical studies have been conducted on IA HA treatment without any other medication in horses [3–7]. Hyaluronan is a glycosaminoglycan component of the synovial fluid (SF) and proteoglycan aggregates in the articular cartilage. The complete mechanism of HA action is not yet fully understood. HA reduces the sensitivity of articular nerve endings in the joint capsule by buffering transmission of mechanical forces to nociceptor nerve endings and thereby reduces pain [8]. HA also reduces impulse activity in the peripheral nociceptor endings by affecting receptor channels [9]. Furthermore, HA has an anti-inflammatory effect [8, 10–12]. However, repeated IA HA injections have also been found to induce a transitional IA inflammation reaction first [13–15], likely caused by low molecular weight breakdown products of HA [14].

Inflammatory and degenerative activity inside the joint can be studied in vivo via analysis of SF biomarkers, which are molecular markers of inflammatory processes and tissue turnover. Research into the biomarkers of equine SF has been focused on finding tools for early diagnosis and monitoring the progression of OA [16–21]. Furthermore, equine SF biomarkers have been used to measure responses to treatments [22, 23] and to evaluate any possible deleterious effects of IA medications [24, 25] and autologous platelet-rich plasma [26].

The aim of this study was to measure SF prostaglandin E_2_ (PGE_2_), substance P, aggrecan chondroitin sulfate 846 epitope (CS846), and carboxypeptide of type II collagen (CPII) concentrations before and 2 weeks after high-molecular-weight non-animal stabilized hyaluronic acid^1^ (NASHA) or placebo (saline) injections in horses suffering clinically from pain in the metacarpophalangeal or metatarsophalangeal joints.

Prostaglandin E_2_ (PGE_2_) was chosen since it is the most important eicosanoid in joint diseases. Elevated SF PGE_2_ levels and increased lameness in horses have been shown in several experimental studies after induction of synovitis or OA [23, 27–29]. Substance P is a pro-nociceptive neuropeptide [30] that has been shown to increase in osteoarthritic equine joints [21]. Furthermore, a relationship between elevated SF substance P concentration and pain and lameness in horses has been reported [31]. In the present study, substance P was chosen because in the first part of the study [32] joint pain and lameness and their response to NASHA^1^ and placebo injections were examined.

The marker of collagen II synthesis has been shown to increase for a prolonged period in the SF of the LPS-induced synovitis model [28]. In contrast to that model and induced OA [17], joint pain was not related to SF CS846 or CPII and cartilage turnover in clinical patients [31]. However, CS846 and CPII were chosen as cartilage turnover markers for the present study.

In contrast with the short half-life of commonly used unmodified HA preparations [33, 34], NASHA has a long half-life (up to 32 days) [35] and remains in the synovial structures for a considerably longer period than other HA products [36–38]. This medication is thus intended for treating human arthritic conditions and potentially has a long-acting clinical effect on relieving pain and inflammation [39]. The pain-relieving effects of NASHA were also recently investigated clinically in lame horses [32]. Our hypothesis was that the decrease of selected SF biomarker concentrations would be greater in the NASHA group horses compared with the placebo group horses.

## Methods

Horses were presented to the University of Helsinki Veterinary Teaching Hospital for the NASHA study. The study was approved by the Viikki Campus Research Ethics Committee of the University of Helsinki. All horse owners signed a study consent before the start of the study and owners were allowed withdraw from the study without giving any particular reason. Twenty-eight horses were included in the present trial.

The NASHA study was performed as a randomised double-blind and placebo-controlled trial with a parallel group design and equal allocation ratio [40]. Before starting the study, a non-blinded assisting technician created a computer-generated randomization list. Horses fulfilling the inclusion criteria were assigned to the treatment (NASHA) or control group according to the randomization list. The list and the NASHA^1^ and placebo products were kept in a locked safe that only the non-blinded assisting veterinarian and the associated technician could access. The NASHA study was planned according to the CONSORT statement and the results of the clinical study have been reported earlier [32].

Horses with lameness due to synovitis of the metacarpophalangeal or metatarsophalangeal joint with or without mild OA and a positive response to diagnostic IA anaesthesia of the affected metacarpophalangeal or metatarsophalangeal joint but with no radiographic signs of OA (subchondral bone lucency or sclerosis, periarticular osteophyte formation, or narrowing of the joint space) of the affected joint were included in the study. Horses with severe or chronic OA were excluded. Mild remodelling of the joint and synovitis of all durations were acceptable. Adult, non-geriatric (age between 4 and 17 years), Finnhorse, Standardbred, Warmblood horses as well as large ponies (height 140–148 cm) of all disciplines were eligible. The ponies are referred to as horses in the text. In addition, horses with IA osteochondral or other fragments were excluded. Bilaterally lame horses and horses that had received IA medications (such as corticosteroids or HA within the previous 3 months or peroral NSAIDs within 15 days) were not eligible. Furthermore, horses with concurrent pathologies (such as clinically significant ligament, tendon, or other soft tissue injuries in the affected limb) were excluded. Treatment groups were equal at the first clinical (baseline) examination; no statistically significant difference between the two groups was found in signalment, use of the horse, radiograph findings, or clinical outcome measures. These measures included lameness, effusion of the affected joint, flexion test result, or pain in flexion. The pain score was created by the authors and recorded as follows: 0 = no pain on flexion, 1 = mild pain (i.e., the horse shows some reaction, such as moving the limb), 2 = moderate pain (i.e., the horse retracts the limb repeatedly during the 1-min flexion period), 3 = severe pain (i.e., the flexion test cannot be properly performed) [32].

The previously published clinical section [32] of the NASHA study provides more detailed data of the study population. Horses were subjected to a full lameness examination and radiographic examination of the affected joint with four standard views as described [32]. Prior to IA anaesthesia (mepivacaine hydrochloride^2^) of the metacarpophalangeal or metatarsophalangeal joint, which was performed to localize the source of lameness and to decide if the horse was eligible for the study (80–100% amelioration of lameness), 5 ml of SF was aspirated into a sterile 5-ml syringe for biomarker measurements. Arthrocentesis was performed through the lateral sesamoidean ligament and blood contamination was recorded if observed in the SF sample. The SF sample was immediately divided into a plain tube on ice (4 ml) and an EDTA (1 ml) tube. White blood cell (WBC) count and protein concentration measurements were performed from the fresh sample in the EDTA tube. Within 1 h of collection, the plain sample was centrifuged at 1700 g for 10 mins in 4 °C, after which it was aliquoted and stored at − 80 °C. If the horse fulfilled the inclusion criteria after examination, the horse received IA NASHA^1^ (3 ml, 20 mg/ml into the affected joint) or an IA placebo injection (3 ml of a sterile 0.9% saline solution^3^) on the same day by the non-blinded assisting veterinarian. The horse was then sent home with the owner and asked to return in 2 weeks, when it was subjected to a second clinical examination [32]. Following the first clinical examination and treatment, the horse was allowed 30 min hand-walking per day and free access to a small paddock during the 2 weeks, after which the second clinical examination was performed and a second SF sample (5 ml) was collected and processed using the same techniques as described above.

SF samples were digested with 0.5 mg/ml hyaluronidase from bovine testes for 30 min at 37 °C prior to analyses of SF PGE_2_, substance P, CS846, and CPII.

### Prostaglandin E_2_

Prostaglandin E_2_ was measured using a commercial enzyme immunoassay kit.^4^ Sample extraction was performed according to the manufacturer’s instructions prior to the immunoassay. Briefly, SF samples were acidified by the addition of 1 M hydrochloric acid to pH 3.5 and then vortexed and centrifuged for 2 min at 12000 × g. The samples were applied to a C18 cartridge^5^ previously conditioned with ethanol and water, and then flushed with water, 15% ethanol, and finally with hexane. PGE_2_ was eluted from the cartridge with ethyl acetate and stored as an elution solution at − 80 °C until analysis. The extracted samples were analysed within 1 week. At the time of the assay, the samples were evaporated to dryness in a vacuum evaporator and reconstituted with assay buffer and analysed according to the manufacturer’s instructions. The samples were extracted and analysed in duplicate. Parallelism was tested for dilutions between 1:2 and 1:50. An appropriate dilution was made for every sample. The detection range was 7.81 to 1000 pg/ml with a detection limit of 11 pg/ml. Recovery of the extraction was 81 to 114%. The intra-assay coefficient of variation (CV) was 14.6%.

### Substance P

Substance P was measured using a commercial enzyme immunoassay kit^6^ according to the manufacturer’s instructions. Samples were analysed in duplicate. The detection range was 3.9 to 500 pg/ml with a detection limit of 10.5 pg/ml. The intra-assay CV was 12.5%.

### Aggrecan chondroitin sulfate 846 epitope

CS846 epitope was measured using a commercial enzyme immunoassay kit^7^ according to the manufacturer’s instructions. The samples were then diluted 1:50 and added to plates in triplicate. The result was reported as the mean of triplicate values, unless the CV was over 20%; in this case the mean of the two closest values was calculated. The detection range was 20 to 1000 ng/ml with a detection limit of 20 ng/ml. The intra-assay CV was < 20%.

### CPII

CPII was measured using a commercial enzyme immunoassay kit^8^ according to the manufacturer’s instructions. The samples were diluted 1:5 and added to plates in duplicate. The detection range was 50 to 2000 ng/ml with a detection limit of 50 ng/ml. The intra-assay CV was 8.1%.

### Statistical methods

A sample size calculator [40] was used with 95% confidence level and 80% power. On the basis of 87% of cases showing clinical improvement in an earlier study on HA for the treatment of naturally occurring arthritic conditions in horses, the estimated sample size was 11 to 14 horses per group [5]. In the placebo group, the proportion that would improve was considered to be 20 to 30%, where 20% is 10% lower than the percentage that has been used in placebo groups in human studies [41, 42].

SF biomarker (substance P, CPII, PGE_2_, CS846) concentrations, WBC count, and protein concentration measurements were analysed with analysis of covariance models. Changes between the baseline and follow-up measurements were used as the response, treatment was used as the fixed effect, and the corresponding baseline measurement as the covariate. For SF concentrations of substance P and CPII and SF WBC count, a logarithmic transformation was used to normalize the distributions. For PGE_2_ concentration an inverse transformation was used to normalize the distributions. CS846 and TP were normally distributed.

The possible effects of background variables (age, gender, breed, use of the horse, lameness score, duration of clinical signs, previous incidences of the affected joint, radiograph findings) on the change in the different biomarker measurements from baseline were assessed with analysis of variance models. The models included the background variable of interest as the sole fixed effect and the change of the biomarker from the baseline as the response. If some of the background variables were statistically significant in these analyses, the effect was also included in the analysis of covariance model for treatment comparison.

We hypothesized that the decrease of these SF biomarker concentrations would be greater in the NASHA group than that of the placebo group. The difference in the change of each SF biomarker concentration after IA injection and a two-sided 95% confidence interval for the difference were estimated from the fitted analysis of covariance models using the contrast method. The estimates of the changes within groups were also calculated. Significance was set at *P* < 0.05 for all analyses. The same statistical analysis software^9^ was used for all statistical analyses.

## Results

Sixty-eight horse owners were interviewed; 36 horses were invited to the first clinical baseline examination. Altogether 30 horses fulfilled the inclusion criteria. Two horses were excluded due to an inadequate amount of high-quality SF for baseline and control samples; 28 horses were thus included in the study. The median age of the horses was 7 years in the NASHA (*n* = 15) and placebo (*n* = 13) groups (range 4–12 and 4–17 years, respectively). There were seven mares, five geldings, and three stallions in the NASHA group. There were six mares, five geldings, and two stallions in the placebo group. Of the horses in the NASHA group, 10 were harness racehorses and five were riding horses predominately used for general-purpose riding. In the placebo group there were seven harness racehorses and six riding horses. The breeds in the NASHA group were Finnhorse (*n* = 8), Standardbred (*n* = 6), and Warmblood (*n* = 1). The breeds in the placebo group were Finnhorse (*n* = 5), Standardbred (*n* = 4), Warmblood (*n* = 2), and pony (*n* = 2). At the first examination, the source of lameness was localized to the right metacarpophalangeal joint in 10 (NASHA group) and seven horses (placebo group), to the left metacarpophalangeal joint in four (NASHA group) and six horses (placebo group), and to the left metatarsophalangeal joint in one horse (NASHA group). No adverse effects of IA NASHA or placebo injections were observed at the second clinical examination or reported by the horse owners.

Background variables (age, gender, breed, use of the horse, lameness score, duration of clinical signs, previous incidences of the affected joint, radiograph findings) did not have any effect on the concentration changes of the different biomarkers.

**Fig. 1:**
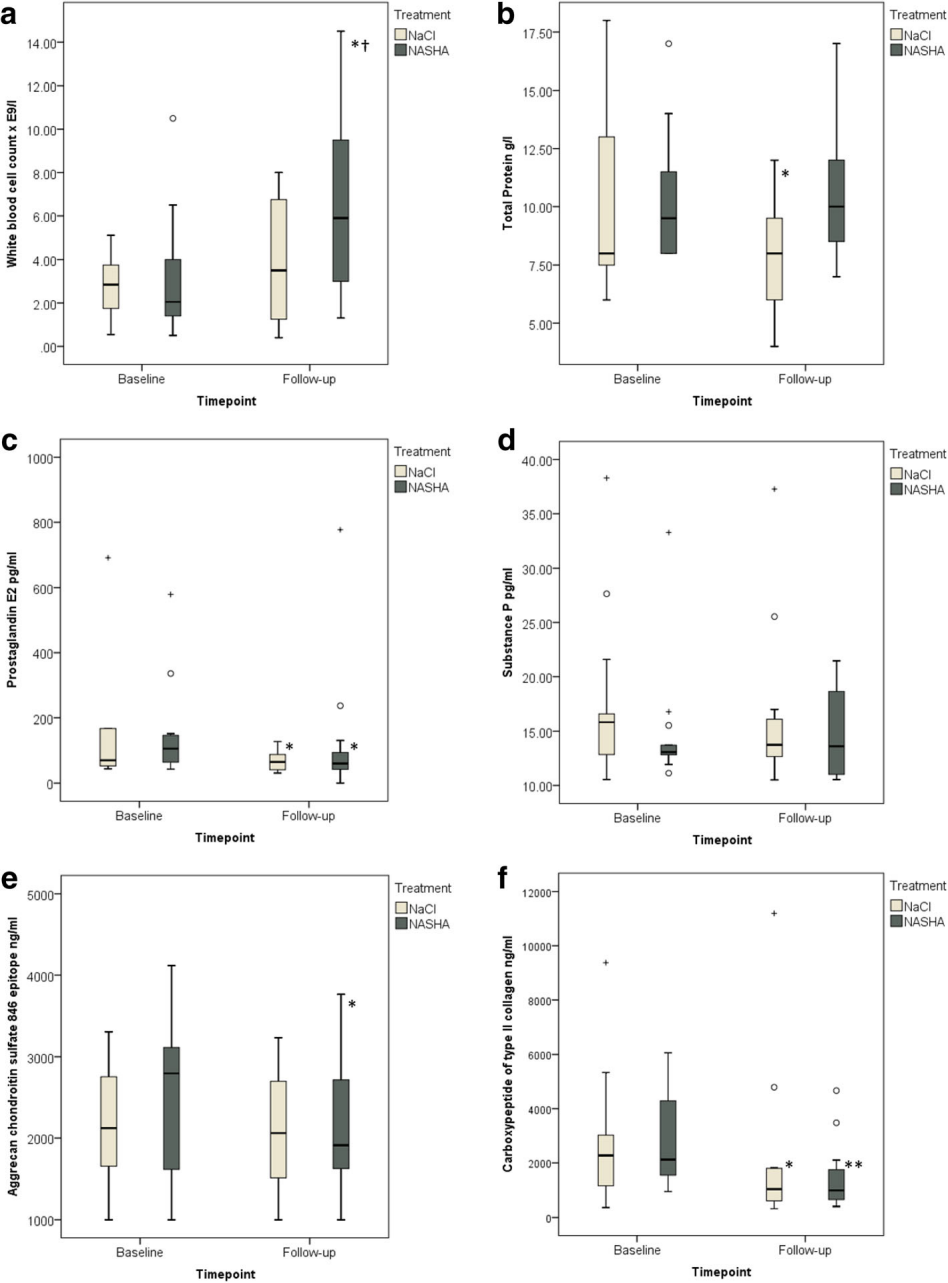
Synovial fluid white blood cell count (**a**), concentrations of total protein (**b**), prostaglandin E_2_ (outliers > 1000 pg/ml are removed to clarify figure) (**c**), substance P (**d**), aggrecan chondroitin sulfate 846 epitope (**e**) and carboxypeptide of type II collagen (**f**) before and after intra-articular NaCl (placebo) or non-animal stabilized hyaluronic acid (NASHA) injection. First and third quartiles are defined by the boxes and median by the band inside. The 2.5th and the 97.5th percentiles are shown as the lower and upper whiskers. *P < 0.05, **P < 0.001 between first and second sample in NaCl (placebo) and NASHA group respectively, † *P* < 0.05 NaCl (placebo) and NASHA groups

NASHA induced a significant increase in SF WBC count when compared with baseline (*P* = 0.021) and the placebo group (*P* = 0.045) (Fig. 1). When the change in SF biomarker concentrations was compared between groups, no significant difference was detected (PGE_2_, *P* = 0.768; substance P, *P* = 0.662; CS846, *P* = 0.207; CPII, *P* = 0.155) (Fig. 1). When examining SF biomarkers within groups, both PGE_2_ and CPII concentrations decreased (PGE_2_, *P* = 0.010 for the NASHA group; *P* = 0.027 for the placebo group; CPII, *P* < 0.001 for NASHA group; *P* = 0.009 for placebo group) (Fig. 1). However, for SF CS846 concentration, a statistically significant reduction from baseline was seen in the NASHA group (*P* = 0.010) but not in the placebo group (*P* = 0.390) (Fig. 1).

## Discussion

In this study, the SF concentrations of selected biomarkers were measured before and 2 weeks after IA NASHA treatment in horses with lameness localized to the metacarpophalangeal or metatarsophalangeal joint.

IA NASHA caused an increase in SF WBC count. Blood contamination during sampling is an unlikely explanation since the arthrocentesis was performed through the lateral sesamoidean ligament, which has been shown to induce less synovial haemorrhage than arthrocentesis through the proximal palmar pouch [43]. In addition, no blood contamination in the analysed SF samples was observed. It seems more likely that IA injection of NASHA induces a mild inflammatory response; this has also been reported after a combined injection of IA pentosane polysulfate and glucosamine injection [44], after repeated IA amikacin injection [45], and after IA gentamicin injection [46]. Moreover, arthrocentesis alone [46] and IA saline injection [44, 45] have been shown to cause significant increases in the SF WBC count. In humans, a transient IA inflammation has been reported after consecutive HA injections [13–15], likely caused by a cell-mediated hypersensitivity reaction [15] to the low-molecular-weight breakdown products of HA [14]. However, in the present study, no clinical signs (such as effusion or pain in the injected joint) were reported by the horse owners or trainers immediately after the injection. Moreover, in most of the samples the increased WBC count was within the normal reference range (< 0.5 × 10^9^/l) or very close (< 1.0 × 10^9^/l) [47]. One explanation for the difference in the WBC count between groups could be decreased SF volume after NASHA injection, although no difference in the joint effusion score between groups was found [32].

When compared with the placebo group, the NASHA injection had no significant effect on SF PGE_2_ concentration. The effect of various substances on PGE_2_ concentration in equine SF has been evaluated previously mainly in experimental studies using induction models [22, 23, 27–29] or horses free of lameness or joint disease [24]. Only Carmona et al. [48] have studied SF markers and their response to treatment in clinical equine patients. As in the present study, they did not find a significant decrease between groups in the SF PGE_2_ concentration when an oral HA formulation was evaluated in horses suffering from osteochondrosis.

PGE_2_ concentration has been shown to increase in SF of equine osteoarthritic joints [19, 21]. Bertone et al. [18] reported SF PGE_2_ concentration to be a good or excellent predictor of joint disease and thus a functional screening test tool in horses. However, high variability in concentrations limited discrimination among the types of joint disease. In contrast, Frisbie et al. [49] speculated that SF PGE_2_ concentration may not be a true indicator of overall joint health, as no remarkable disease-modifying effects were detected after phenylbutazone treatment in their experimentally induced equine OA model despite decreased PGE_2_ concentration. In the present study, we were unable to observe significantly decreased SF biomarker concentrations in the NASHA group compared with placebo. This may be a result of high variation in concentrations initially, and more specifically, high variations in responses to the treatment. In the case of synovitis, PGE_2_ concentration may decrease more rapidly than in mild OA. Frisbie et al. [17] showed that there is a long-standing increase in PGE_2_ concentration after arthroscopically induced OA while the increase was short-term in other studies using the LPS inflammation induction model [28, 29]. However, the lack of treatment effect by HA on SF PGE_2_ concentration was also reported in experimental studies with more uniform osteoarthritic joints [12, 22]. Another study by de Grauw et al. [31] concluded that PGE_2_ concentration might reflect joint pathology in general rather than specifically pain arising from the joint.

In the present study, no differences in SF substance P concentrations were observed within or between treatment groups. This is in contrast with the reported elevated concentrations in SF from osteoarthritic joints compared to normal equine joints [21]. Substance P concentration has been shown to be associated with joint pain [31]. In the equine LPS-induced synovitis model, substance P decreased following meloxicam treatment [28] but not after IA opioid analgesia [23]. Both medications resulted in decreased lameness. The differences in responses after the aforementioned medications can be explained by different mechanisms of action (i.e., opioid analgesia has presynaptic control while NSAIDs influence the release of substance P) [28, 31]. The complete mechanism of action of HA in the joint is not known but it has been shown that HA reduces the sensitivity of articular nerve endings by buffering transmission of mechanical forces to nociceptor nerve endings [8] and by affecting pain receptor channels [9]. The binding of neuropeptides could be one pain-reducing mechanism of HA. The results of the present study suggest, however, that intrasynovial NASHA injections do not have an effect on SF substance P concentration. The clinical section of this NASHA study showed reduction in pain sensation, as horses had a statistically milder response in the flexion test after they had received an IA NASHA injection [32]. This may be due to reducing sensitivity of nerve endings by the aforementioned mechanisms. However, the lameness score was not significantly different between the NASHA and placebo groups [32].

Frisbie et al. [17] showed a significant increase of SF CS846 and CPII concentrations in horses with induced OA compared to exercise-only horses. Although the change in SF CS846 concentration was not different between the treatment groups, the concentration decreased in the NASHA group after the IA injection in the present study. CS846 concentration has been shown to increase in SF following naturally occurring injury and OA in humans [50] and after surgically induced OA [51] and LPS-induced synovitis [52] in horses. Furthermore, exercise induces elevation of SF CS846 concentration in horses [17].

CS846 levels were reported to have a significant correlation with CPII [17]. However, such a correlation was not shown when the effects of meloxicam on cartilage turnover was studied in the equine LPS-induced synovitis model. While no treatment effect was seen on CS846 epitope levels, CPII concentration was significantly reduced compared to placebo [28]. In addition, phenylbutazone has been shown to reduce CPII levels in a comparable equine model [53]. Along with the increase of CPII, an increase in CS846 after IA lidocaine and bupivacaine injections in healthy equine joints was speculated to be the reparative response to the short chondrotoxic effect of these compounds [24]. As IA mepivacaine was injected on the same day (NASHA was also injected on the same day), it is possible that this IA anaesthetic was interfering with the biomarker concentrations to some extent. IA methylprednisolone was reported to elevate CS846 in horses free of joint disease, which suggests an increase in articular matrix turnover [25].

The cause for the decrease in CPII is unclear. SF CPII concentration decreased in both groups compared with pre-injection samples. Although the decrease could be interpreted as impaired synthesis of type II collagen, it may also reflect the fact that less damage to collagen has occurred in the joint after medication [28]. Unfortunately, the collagen degradation marker C2C was not measured in the present study to demonstrate this. It has been shown that CPII increases in equine joints after different types of insults, such as after LPS injection in the normal equine intercarpal joint [52] and after exercise or surgically induced OA [17]. The decreasing concentrations of SF CPII after IA injections in the present study could indicate compromised synthesis of type II collagen. This may also indicate less degradation of cartilage after the horses had discontinued training and rested due to participating in the study.

A 2-week period between SF sampling was chosen to optimize the clinical action of the NASHA injection [32]. The maximum effect of pain reduction of IA HA injection has been shown to be between 2 to 6 weeks post-treatment [7, 39]. On the other hand, some of the SF markers could have returned to baseline even earlier regardless of NASHA or saline injection. In the equine LPS induction model, PGE_2_ and CS846 were reported to return to the baseline level before or at 168 h post-injection, while CPII stayed above the baseline level after that [52]. However, since our study population consisted of client-owned patients and many had to travel a long distance to the hospital, it was logistically difficult to organize additional SF sampling visits, either between the baseline and the final samplings or after 2 weeks. Frequent sampling of the SF also induces articular inflammation and therefore has an effect on the measured parameters, which would make assessment of these results challenging.

The absence of significant differences in the SF markers between groups could due to the wide variations in concentrations within groups. The intra-assay CV was also relatively high in some analyses (> 10% for PGE_2_, substance P, and CS846). The disease stage and the intensity of inflammation influence concentrations of cartilage-derived markers [54]. Furthermore, time and type of primary insult, duration of lameness, disease, and the extent of joint effusion may have an effect on the SF concentrations of different markers. However, these variables were controlled as best as possible in the present study, as the demographic and clinical variables were similar between the treatment groups [32]. Also, demographic and clinical variables had no effect on the SF biomarker concentrations. Despite these precautions, inflammation may have been more acute and cartilage matrix degradation more advanced in some of the joints. The high variation in the initial SF baseline biomarker concentrations reflects this. Therefore, although we had calculated the group size according to previous studies, a much larger sample size is necessary to show possible differences between the groups.

## Conclusions

Although cartilage-derived biomarker CS846 concentrations in SF decreased only in the NASHA group, no statistically significant change of any of the biomarkers was observed between the two treatment groups in this study. The IA NASHA injection induced significant increase in SF WBC count; this may indicate an inflammatory response akin to that also reported in human studies. However, IA NASHA injection was well tolerated, as no clinically adverse effects were observed.

## Abbreviations

CPII: Carboxypeptide of type II collagen
CS846: Aggrecan chondroitin sulfate 846 epitope
HA: Hyaluronan
IA: Intra-articular
NASHA: Non-animal stabilized hyaluronic acid
OA: Osteoarthritis
PGE_2_: Prostaglandin E_2_
SF: Synovial fluid
WBC: White blood cell

## Footnotes

1 Durolane, Bioventus LLC, Durham, NC, USA

2 Scandicain, Astra Zeneca, Cambridge, UK

3 Sodium chloride 9 mg/ml, B Braun, Germany

4 PGE_2_ high sensitivity EIA kit, Enzo Life Sciences, Plymouth Meeting, PA, USA

5 Sep-Pak, Waters, Milford, MA, USA

6 Cayman Chemical, Ann Arbor, MI, USA

7 IBEX Pharmaceuticals Inc., Montreal, Quebec, Canada

8 IBEX Pharmaceuticals Inc., Montreal, Quebec, Canada

9 SAS System for Windows, version 9.3, SAS Institute Inc., Cary, NC, USA
